# Prebiotic, Antipathogenic Bacteria and Hypocholesterolemia Properties of Fermented Rice Bran Extracts Derived from Black Rice and Germinated Brown Rice

**DOI:** 10.3390/foods11223704

**Published:** 2022-11-18

**Authors:** Khaetthareeya Sutthanut, Patcharaporn Tippayawat, Sukanya Srijampa, Wisitsak Phoksawat, Pornchanan Vachirodom, Roongrawee Wandee

**Affiliations:** 1 Department of Pharmaceutical Chemistry, Faculty of Pharmaceutical Sciences, Khon Kaen University, Khon Kaen 40002, Thailand; 2 Human High Performance & Health Promotion Research Institute: HHP&HP Research Institute, Khon Kaen University, Khon Kaen 40002, Thailand; 3 Department of Medical Technology, Faculty of Associated Medical Sciences, Khon Kaen University, Khon Kaen 40002, Thailand; 4 Department of Microbiology, Faculty of Medicine, Khon Kaen University, Khon Kaen 40002, Thailand

**Keywords:** rice bran, black rice, germinated brown rice, ferment, prebiotic, antibacterial, toxicity

## Abstract

Rice bran is a rich source of health-promoting nutrition and bioactive compounds; nevertheless, the properties of rice brans depend on cultivars, ages, and preparation methods, drawing the potential of raw materials for health benefits. Therefore, this research aimed to investigate the health-promoting properties of fermented rice bran extracts from cultivar black rice (H7F) and germinated brown rice (G13F), focusing on their prebiotic, antipathogenic bacteria activity and safety demonstrated in vitro and in vivo study models, respectively. Here, the screening of metabolites’ change after rice bran fermentation by ATR-FTIR spectra revealed specific peaks corresponding to the composited components of protein, carbohydrate, and lipid. Then, in the in vitro study, the prebiotic capability of H7F and G13F extracts was demonstrated by a growth-promoting effect on *Lactobacillus delbrueckii* subsp. *lactis* under specific acidic conditions. Furthermore, antipathogenic bacterial activity against *Escherichia coli* and *Staphylococcus aureus* was presented at 25 mg/mL of MIC values and 50 mg/mL of MBC of both fermented rice bran extracts, eliminating the bacteria by interfering with the biofilm formation. For safety, an acute and chronic toxicity study using Wistar rats was conducted, in which changes in the body and organ weights, histopathology of organs, blood chemistry, and hematological parameters were observed after H7F and G13F treatment. Desirably, they showed no toxicity, with a significant reduction in blood cholesterol levels in the chronic treatment of H7F and G13F. Conclusively, the overall results evidenced the health benefits of H7F and G13F related to their prebiotic and antipathogenic bacteria properties and hypocholesterolemia potential with a high level of safety. Therefore, the fermented rice bran extracts were demonstrated as potential materials for the further development of functional ingredients and health products.

## 1. Introduction

Rice is one of the most important crops cultivated worldwide, especially in the Asian region [1,2]. Thailand is one of the top 10 rice-producing countries and a banner exporter of processed rice products [3]. Rice bran, a rice milling by-product, is a nourishing source of phytochemical compounds, including oryzanol, γ-amino butyric acid (GABA), phenolics, flavonoids, arabinoxylan, phytosterols, phytic acid, and vitamins, etc. [4,5,6,7]. According to several previous studies, rice bran extracts from various kinds of colored rice (pink, red, purple, brown, and black) have been proven to contain health-promoting benefits, e.g., antioxidation, anti-inflammation, and anticancer effects, as well as the improvement of lipid profiles [6,8,9].

Prebiotics and probiotics are functional health-promoting food ingredients on demand in the market with highly safe and naturally health-balancing features. Prebiotics are nondigestible foods that can enhance the propagation and functions of health-beneficial gut microbiomes—probiotics such as *Bifidobacterium* and *Lactobacillus* spp. [10], the two common genera of probiotic bacteria, with some isolated species: *L. plantarum*, *L. casei*, *L. acidophilus*, *B. animalis*, and *B. bifidum*. Probiotics have antipathogenic activity associated with producing bioactive compounds, short-chain fatty acids (SCFA), peptides, and bacteriocins. Prebiotics possess health-promoting effects by modulating the immune system, lowering allergy risk, reducing the production of harmful protein metabolites, and suppressing pathogenic bacterial growth in the gastrointestinal tract [11,12]. Prebiotics such as dietary fiber are food components that enhance the growth of probiotic bacteria; for instance, the fructooligosaccharides found in onion, asparagus, and wheat are the source of *Bifidobacterium* probiotics [12]. In addition, combining prebiotics and probiotics, known as synbiotics, offers intensive synergistic benefits on multiple health issues: nutrition, preventing infant diseases, reducing irritable bowel syndrome-relating symptoms and compromising bacterial vaginosis and urinary tract infection, and lowering cholesterol blood level [12,13,14,15]. 

Interestingly, the prebiotic and synbiotic properties of rice bran extracts have been evidenced. Dietary rice bran enhanced the growth and colonization of *Lactobacillus rhamnosus* GG and *Escherichia coli* Nissle 1917 [16], which was effective against human rotavirus (HRV) diarrhea, promoted maintenance of gut epithelial health during infection, and improved innate immune response by increasing intestinal IFN-γ and total IgA level in gnotobiotic pigs [17]. Moreover, fermented rice bran by *Lactobacillus plantarum* B2 featured a synbiotic product in promoting fecal lactic acid bacteria growth and inhibiting pathogens (*Escherichia coli* and *Salmonella* spp.) in Wistar rat intestine [18]. Interestingly, synergistic effects from combinational treatment of Thai black rice bran extract and yeast beta-glucan showed an increase in antioxidant capacity and reduced inflammatory cytokines in colitis-induced rats [6,19]. Moreover, fermentation is a biochemical process to improve rice and rice bran’s nutritional quality and biological activities [20,21]. Fermentation increased crude protein contents, phytochemicals, and gamma-butyric acid in fermented rice bran [21,22]. 

Although many prior studies have reported various health benefits of rice bran extracts, there is very little information regarding fermented rice bran from KKU ULR0381 black rice cultivar (H7F) and germinated brown rice (G13F). Thus, this study aimed to investigate the biochemical components of fermented rice bran extracts by attenuated total reflection–Fourier transform infrared spectroscopy (ATR-FTIR), the prebiotic property promoting *Lactobacillus dellbrueckii* (*L. dellbrueckii*) subsp. *lactis* growth, antipathogenic bacteria activity, and a safety and toxicity test in both in vitro and in vivo models. The obtained information will deliver a guideline to support health product development from H7F and G13F. 

## 2. Results

### 2.1. Comparison of Biochemical Components between Nonfermented and Fermented Rice Bran by Derivative FT-IR Spectra

**Figure 1:**
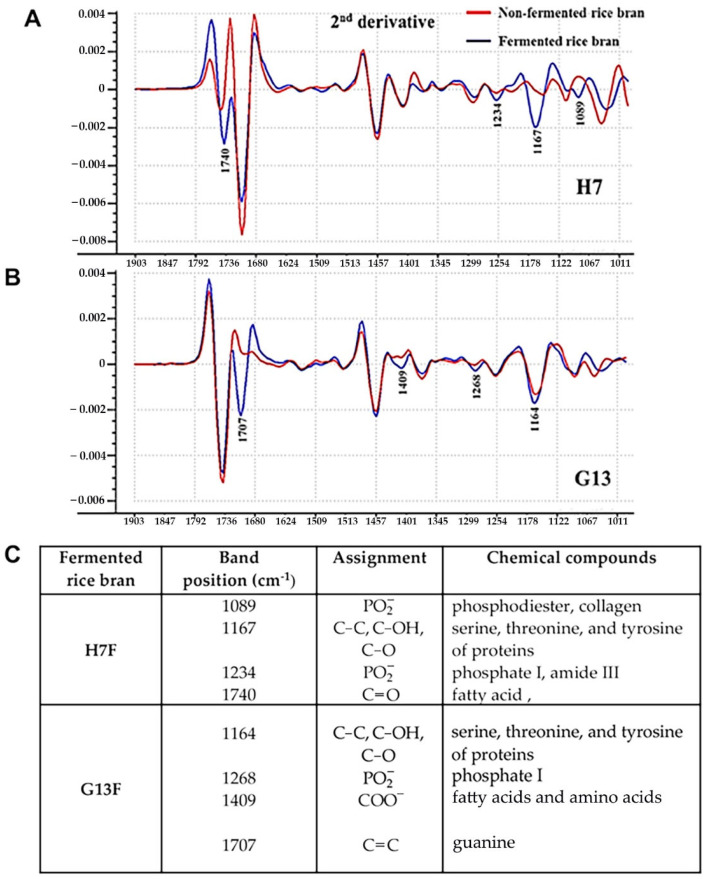
Comparison of the second derivative spectra between H7NF vs. H7F (**A**) and G13NF vs. G13F (**B**) by ATRFTIR. The prediction of biochemical compositions’ change by using the band positions at specific wavelength number (cm^−1^) of IR spectra of H7F and G13F compared to H7NF and G13NF, respectively (**C**).

In this study, ATR-FTIR was used to monitor two different fermentative rice brans compared with nonfermentative forms of those rice brans. The second derivative ATR-FTIR spectra of H7F and G13F showed different compositions of biochemical ingredients from nonfermented rice bran (H7NF and G13NF) (Figure 1). Both fermented rice bran extracts exhibited feature peaks corresponding to protein, carbohydrate, and lipid composition. For the H7F sample, the specific peaks included phosphate and collagen at wavelength number position 1089 cm^−1^, serine, threonine, and tyrosine of proteins at 1167 cm^−1^, phosphate I and amide III at 1234 cm^−1^, and fatty acid at 1740 cm^−1^ of the H7F spectrum (Figure 1A). On the other hand, at the G13F spectra, the major peaks were found at 1164 cm^−1^ (serine, threonine, and tyrosine of proteins), 1268 cm^−1^ (phosphate I), 1409 cm^−1^ (fatty acid and amino acid), and 1707 cm^−1^ (guanine). The detailed information on biochemical compositions’ change after fermentation is shown in Figure 1C.

### 2.2. Lactic Acid Bacteria Growth Promoting of Fermented Rice Bran Extracts 

**Figure 2:**
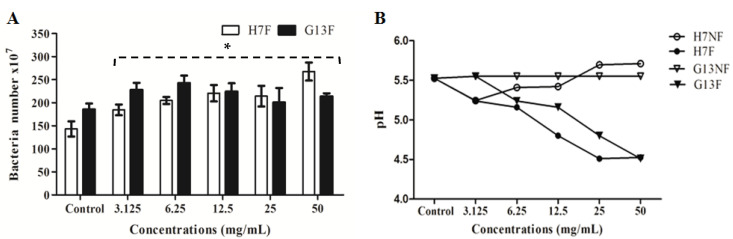
The fermented rice bran extracts (H7F and G13F) promoting *L. delbrueckii* subsp. *lactis* growth determined by flow cytometry compared to the control (**A**) and a pH gradient during various concentrations of nonfermented rice bran extracts (H7NF and G13NF) and fermented rice bran extracts (H7F and G13F) (**B**); * significant difference when compared to the control (*p*-value < 0.05).

According to the metabolites’ change of rice bran under the fermentation, the fermented rice bran extracts (H7F and G13F) were used to prove the prebiotic property for a probiotic bacterium—*L. delbrueckii* subsp. *lactis*. To determine whether H7F and G13F extracts can promote the growth probiotic bacteria in in vitro study, the numbers of *L. delbrueckii* subsp. *lactis* were determined after culture with the extracts. The probiotic was significantly increased in coculture condition of all concentrations (3.125, 6.25, 12.5, 25, and 50 mg/mL) of both H7F and G13F extracts (Figure 2A), and it can observe the plateau in the numbers of lactic acid bacteria at concentrations of 12.5 mg/mL of the G13F extract. Moreover, the pH of nonfermented rice bran extracts (H7NF and G13NF) and fermented rice bran extracts (H7F and G13F) were evaluated to indicate optimal acid production to promote the lactic acid bacteria growth. The reduction in pH values, the higher level of lactic acid production (Figure 2B) with a dose-dependent trend (decreasing pH) depending on the extract concentrations. The results supported that the fermented rice bran extracts (H7F and G13F) could promote *L. delbrueckii* subsp. *lactis* in the apparent optimal growth at acidic pH; optimal pH range between 4.5–5.2.

### 2.3. Pathogenic Bacteria Growth Inhibition of Nonfermented and Fermented Rice Bran Extracts 

**Figure 3:**
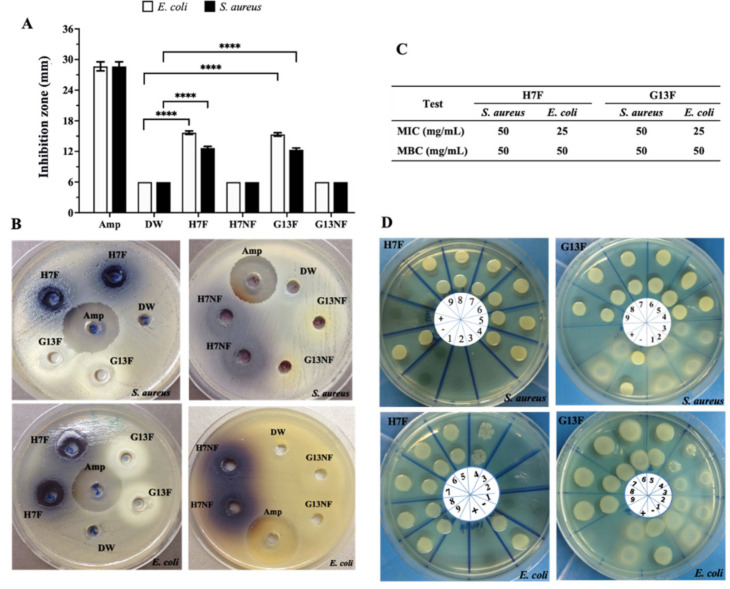
Screening of antimicrobial effects of fermented rice bran H7 (H7F) and G13 extract (G13F) compared to nonfermented rice (H7NF and G13NF). According to the well diffusion assay, only H7F and G13F significantly exhibited inhibition zone against *Escherichia coli* and *Staphylococcus aureus* (**A**,**B**). A minimum inhibition concentration (MIC) and minimum bactericidal concentration (MBC) values were also evaluated by broth microdilution technique (**C**,**D**). Various concentrations of H7F for MIC and MBC were investigated, as follows: lane 1 = 200 mg/mL; 2 = 100 mg/mL; 3 = 50 mg/mL; 4 = 25 mg/mL; 5 = 12.5 mg/mL; 6 = 6.25 mg/mL; 7 = 3.12 mg/mL; 8 = 1.56 mg/mL; 9 = 0.8 mg/mL, respectively. Distilled water (DW) was used for noninhibition control (or lane +), and 5 μg/mL of Ampicillin drug (Amp) was used for inhibition control (or lane −). **** indicates statistical significance at *p*-value < 0.0001.

The antibacterial potential of nonfermented and fermented rice bran extract against pathogenic bacteria was evaluated by agar well diffusion assay. The 400 mg/mL concentration of either H7F or G13F extracts inhibited the growth of both *S. aureus* and *E. coli*; the averages of the inhibition zone of H7F were 12.15 and 15.96 mm, and of G13F were 12.15 and 15.50 mm, respectively. Unlike the fermented rice bran extracts, the nonfermented rice bran samples (H7NF and G13NF) could not inhibit these two pathogenic bacteria (Figure 3A,B). In addition, the results from the broth microdilution technique showed equivalent degrees of antimicrobial activities between H7F and G13F samples. The minimum inhibitory concentration (MIC) values of H7F and G13F on *E. coli* and *S. aureus* growth were identically detected at 25 and 50 mg/mL concentrations, respectively. For minimum bactericidal concentration (MBC), 50 mg/mL of both fermented rice bran extracts could inhibit the growth of *S. aureus* and *E. coli* (Figure 3C,D). 

### 2.4. Effect of the Fermented Rice Bran on Biofilm Formation

**Figure 4:**
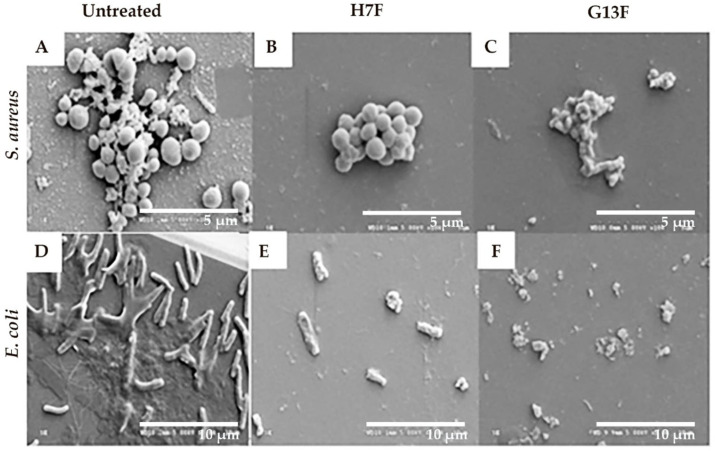
Fermented rice bran H7 (H7F) and G13 extract (G13F) inhibited biofilm formation. However, biofilm and colonization of *Staphylococcus aureus* were still observed in the untreated condition (**A**), while they were decreased in the treated groups with either H7F (**B**) or G13F extracts (**C**). In addition, while the control (untreated) group of *Escherichia coli* still presented biofilm formation, flagella structure, and colonization (**D**), they were decreased in the treated groups with either H7F (**E**) and G13F extracts (**F**). Interestingly, cell membrane breakage of *E. coli* (a Gram-negative bacillus) was displayed in the treatment of either H7F or G13F (**E**,**F**).

Following the demonstration of antipathogenic bacterial effects after treatment with the fermented rice bran extracts (H7F and G13F), the inhibitory mechanism against pathogenic bacteria of H7F or G13F was further investigated. Biofilm formation is an important virulence factor produced by both *S. aureus* and *E. coli*; this mechanism can prevent pathogenic bacteria destruction by toxic agents [23,24]. In our study, the H7F and G13F at half of MBC concentration (25 mg/mL) were cultured with *S. aureus* and *E. coli*; consequently, the biofilm formation and bacterial destruction were delineated by imaging the morphology of *S. aureus* and *E. coli* after the sample treatment under a scanning electron microscope (SEM). Biofilm formation reduction and bacterial cell breakage were detected in the treated groups, which differed from the control (untreated) group of each bacteria type. The control group showed colonization and biofilm formation of *S. aureus*; similarly, the control for *E. coli* presented biofilm formation and colonization with a flagella structure. Based on the results, the induction of bacterial cell membrane breakage and colony shrinkage was suggested as a mechanism of antimicrobial activity detected in H7F and G13F (Figure 4). 

### 2.5. Toxicity of the Fermented Rice Bran Extracts In Vitro Study 

**Figure 5:**
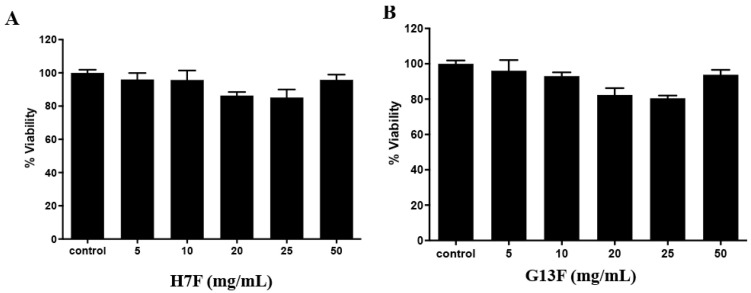
Toxicity test of fermented rice bran H7 (H7F) and G13 extract (G13F) to human PBMCs by MTT assay. Both H7F (**A**) and G13F (**B**) extracts showed no toxicity to human PBMCs, expressing more than 80% of cell viability at all tested concentrations.

After fermentation of the rice brans, we demonstrated the metabolites’ change and acidic increase; thus, before using them as a supplement or functional food/beverage, toxicity to human and animal models should be investigated. The toxicity to human peripheral mononuclear cells (PBMCs) was demonstrated in in vitro study. For the result, all the concentrations of either H7F or G13F extract showed no toxicity to human PBMCs with more than 80% cell viability compared to the control (untreated group) (Figure 5A,B). The %survival of the cells of both fermented rice bran extracts’ treatment might indicate that these metabolic compositions and acidic conditions after the rice bran fermentative process were nontoxic to human PBMCs [25].

### 2.6. Toxicity in Acute or Chronic Manner of the Fermented Rice Bran Extracts In Vivo Study 

To confirm the safety and bioavailability of product use, the study in the animal models should be demonstrated. In our study, Wistar rats were used for toxicity study in the acute or chronic manner. The growth and behavior of all the animals after receiving a single dose of G13F and H7F extracts at each concentration of 1000, 2500, and 5000 mg/kg/day were normal over a 14-day period without detection of mortality, change of hematological and biochemical values, or any pathological lesion of organs.

For chronic toxicity, both the male and female animals that received fermented rice bran H7F or G13F showed healthiness and no signs of toxicity when compared to the control group. In addition, there were no significant differences in body and organ weights among the treated groups that received different doses of the extracts. However, there were changes in WBC, platelet, and cholesterol levels after treatment with specific doses of H7F or G13F. For hematological values, the increase in WBC levels in all treated groups of H7F and G13F of both genders was demonstrated; however, significant differences were detected at high doses; 2000 mg/kg of H7F and 500 mg/kg of G13F in female rats as well as of 1000 and 2000 mg/kg of G13F in male rats, when compared to the vehicle group (Table 1). Furthermore, compared to the untreated group, higher platelet count levels of male rats receiving 500, 1000, and 2000 mg/kg of G13F extract were significantly revealed (Table 1). Additionally, the reduction in cholesterol levels in 500 and 2000 mg/kg of H7F and 1000 mg/kg of G13F-treated groups of male rats was significant compared to both the vehicle and untreated groups. In contrast, others presented no statistical significance (Table 2). 

## 3. Discussion

Rice bran—the outer layer fractions of rice grains produced from rice milling—is a rich source of fiber, vitamins, amino acids, and phenolic compounds. The high bioactivity and antioxidative properties of rice are potentially fortified through germination and fermentation [20,21,26,27,28,29]. Therefore, evidence of the genuine benefits of germinated rice and fermented rice and their extracts is pivotal in supporting the rationale for health-promoting applications. The present study has aimed to examine the properties of fermented rice bran of selected rice cultivars, focusing on the screening of biochemical composition by ATR-FTIR, prebiotic properties, antipathogenic effects, safety, and toxicity in both acute and chronic manners. This is to obtain scientific evidence that will serve as guidelines for further developing new functional food supplementary products from these ingredients. 

The technology of ATR-FTIR was selected in this study to enable direct analysis of the chemical composition of the sample based on a total internal reflection via evanescent waves [30,31]. The ratios of prominent biochemical compounds, including amino acids (serine, threonine, and tyrosine) and polysaccharides, of fermented-rice bran derived from fermentation by *L. delbrueckii* subsp. *lactis* of both H7 and G13 were increased when compared to nonfermented rice bran samples. The results agreed with those of Russo et al. [32], who reported an increasing level of gross energy and crude protein after the fermentation of rice bran. Macek et al.’s study reported that the fermented rice can produce amino acids such as serine, threonine, and tyrosine. These amino acids are essential factors of protein biosynthesis in organism, including boosting the growth of microorganism [33]. In addition, fatty acid was increased after both rice fermentation. These results are in accordance with previous research that showed a slight increase in fatty acid after fermenting chia and sesame seeds [34]. An increase in fatty acids such as short-chain fatty acids in the fermented product cloud be attributed to microorganisms’ metabolism [35], producing SCFA (acetate, propionate, and butyrate) from carbohydrates and protein catabolism [36,37]. Through biosynthetic and metabolic versatility, fermentation by lactic acid bacteria is a value-added form of food processing to fortify the products with macronutrients, micronutrients (vitamins), or non-nutrition such as bacteriocin and phytochemical derivatives [20,32]. 

To answer whether H7F and G13F extracts could promote the growth and lactic acid production of *L. delbrueckii* subsp. *lactis*, a broth dilution technique was employed. The results revealed that both extracts of fermented rice bran could virtually enhance the propagation of probiotic bacteria. In addition, the acidic property of the fermented rice bran extracts, particularly at pH range 4.5–5.2, could be suitable to promote *L. delbrueckii* subsp. *lactis*, resulting in organic acid production under the growth of lactic acid bacteria. This result has implied the health impact of H7F and G13F following the principle of prebiotic-fortified probiotic functions. Probiotic bacteria in the gastrointestinal tract have various beneficial effects, e.g., maintenance of intestinal microbial balance, production of antimicrobial substances against pathogens, induction of mucin production, stimulation of a heightened immune response, and modulation of cytokine production by intestinal epithelial cells (IECs) [38,39,40]. Therefore, H7F and G13F are likely effective prebiotic agents containing bioactive compounds and nutrients for probiotic microorganism growth promotion. Moreover, the plateau of the *L. delbrueckii* subsp. *lactis* population at a G13F concentration greater than 6.25 mg/mL was demonstrated; this reflected the saturation of the *L. delbrueckii* subsp. *lactis* population in the systems affected by high concentrations of G13F extract (12.5–50 mg/mL) (Figure 2A). This has suggested the high potential and attribution of the feature biochemical composition in G13F—the combination of fatty acids, amino acids, and guanine (Figure 1). 

Furthermore, the bactericidal property of H7F and G13F against pathogenic bacteria (*S. aureus* and *E. coli*)—usually causative microorganisms of intestinal infectious diseases such as acute diarrhea and inflammatory bowel disease (IBD)—was investigated [41]. This study showed that H7F and G13F had a gainful property of inhibiting the growth of these intestinal pathogens, while nonfermented extract (H7NF and G13NF) failed to demonstrate this capability. The antibacterial activities could be attributed to some non-nutrition metabolites, such as bacteriocins, coordinating with low pH conditioning from lactic acid bacteria after fermentation [12]. This presumption is supported by the previous reports. *L. delbrueckii* is a nonmotile Gram-positive rod bacterium that biochemically transforms sugars into lactic acids via anaerobic fermentation; therefore, many subspecies, including *L. delbrueckii* subsp. *delbruecki*, *L. delbrueckii* subsp. *bulgaricus*, and *L. delbrueckii* subsp. *lactis*, have been widely employed in the yogurt, cheese, and dairy products industry. Interestingly, the health benefits of *L. delbrueckii* subsp. *lactis* have been potentiated by several reports on the ability in bacteriocin production—namely lacticin, which has a broad spectrum of activity against Gram-positive bacteria and other microorganisms with high tolerance to intestinal antimicrobial molecules [42,43,44,45]. Moreover, this was supported by the report of Li, Lee, and Cho [24]; the effective microorganism-fermented extract (EM)-YU, which is a new version of the refreshment drink derived from fermented rice bran, seaweed, and kiwifruit, was found to exhibit antibacterial activity against *E*. *coli* O157:H7 and *P*. *aeruginosa* PAO1 under low pH (at 3.5) conditions. However, apart from fermentation extracts, nonfermented rice bran extracts of Song-Yod and jasmine rice from different extraction techniques also reported their antimicrobial activity against *Salmonella* spp., *Shigella* spp., *E*. *coli*, *V*. *cholera*, *V*. *vulnificus*, and *S*. *aureus* [41]. Consequently, a further suggestion from the present study is that the efficiency of the antibacterial activity of rice bran may depend on other factors such as rice species, the extraction method of rice bran, and the strain of bacteria. 

Biofilm is an extracellular polymeric conglomeration of shading proteins, capsular polysaccharides, and slime covering the surface of their colonization. It is a naturally protective armor and contributes to the encouraging environment for bacterial growth [46,47]. In this study, biofilm formation, observed by SEM, in both *S. aureus* and *E. coli* reduced remarkedly after treatment of either H7F or G13F. Notably, both H7F and G13F could promote breakage of the cell membrane of *E. coli*. As supporting evidence, a biofilm inhibitory effect against *E. coli* O157:H7 by crystal-violet biofilm assay of diluted EM-YU was previously reported [24]. Moreover, Bermudez-Brito et al. [48] reported that lactic acid could destroy Gram-negative bacteria by inducing cell perforation and inhibiting cell membrane procreation. This was attributed to pH decreasing in bacterial cytoplasm and promoting bacteriocin production from lactic acid bacteria [48]. 

Safety and toxicity information on H7F and G13F is necessary to foster the development of new supplementary food products. This present research work is conducted in in vitro and in vivo test models. Human PBMCs were cocultured with fermented rice bran extracts before the percentages of live and dead cells were investigated by MTT assay. It was revealed that all concentrations of H7F and G13F showed more than 80% of cell viability, indicating no cytotoxicity to human PBMCs [25]. Relatively, their safety for acute and chronic administration was significantly agreed upon and evidenced. For acute toxicity, the fermented rice bran extracts showed no toxic effects on body weight, organ weight, blood chemical and hematological values, and histopathological analysis. These obtained results indicated the safety of these extracts in the acute phase at all dosing levels. Moreover, the chronic toxicity test showed no sign of weight loss or pathological lesions of vital organs in either male or female rats. Moreover, PBMC numbers increased significantly in many concentrations of H7F and G13F consumption. Presumably, the increase in innate immune cells (macrophages) may result from the presence of peptidoglycan, which is a part of the lactic acid bacteria cell wall via inducing the secretion of proliferative-associated cytokines [49]. Furthermore, G13F seemed to stimulate platelet production. However, the mechanism at this point remains unclear. 

Interestingly, a significant reduction in cholesterol levels at 500 and 2000 mg/kg of H7F and 1000 mg/kg of G13F in male rats was revealed, compared to those in both vehicle and untreated groups. These results support health homeostasis through hepatic metabolism. Although the amount of short-chain fatty acid (SCFA) increases in prebiotic fermentation, the lipid blood level is maintained through hepatic metabolism of the SCFA as propionate and butyrate from the portal circulation to prevent systemically high SCFA concentrations [50]. Therefore, the contribution of dietary fibers and biochemical constituents from rice bran and lactic acid bacteria is presumed, which can disturb lipid digestion, absorption, and metabolism [11,12]. Dietary fibers can bind to some minor molecules, bile salt, or enzymes, resulting in reduced lipid digestibility and absorption. The binding property of fiber to bile acids, fatty acids, and cholesterol has been suggested as an underlining mechanism [51]. Besides the nutrition, phytochemicals such as anthocyanin-pigmented rice extract (black rice) could partially contribute to decreased plasma triglyceride, total cholesterol, and low-density lipoprotein cholesterol in rats [52]. Additionally, lactic acid bacteria probably disturb lipid metabolism via deconjugating bile salt and suppressing cholesterol absorption, which agrees with the findings of Pereira and Gibson [53]. Germinated brown rice improves lipid parameters and ameliorates cardiovascular disease (CVD) risk via transcriptional regulation of hepatic metabolic genes [54]. The hypocholesterolaemia effect of rice bran in high-fat diet-fed mice has been evidenced in association with the phytochemical composition of oryzanol, anthocyanins, GABA, and vitamin E [55,56]. Interestingly, the nutrition-fortified final products with GABA and phenolics from the processes of fermentation and germination have widely been evidenced [57,58,59,60,61]. Therefore, our study revealed the potential properties of both fermented rice bran extracts derived from black rice and germinated brown rice, including prebiotic properties, antipathogenic bacteria, and safety with hypocholesterolemia effect demonstrated in in vitro and in vivo models, respectively. The obtained information could emphasize that fermented black rice (H7F) and germinated brown rice (G13F) are the potential functional ingredients for the further development of health products such as probiotics, prebiotics, and synbiotics, as well as fermented foods.

## 4. Materials and Methods

### 4.1. Preparation of the Rice Bran Extracts 

Rice bran of Thai cultivar black rice (H7; KKU ULR0381) and germinated brown rice (G13) were purchased from local rice farmers in Khon Kaen province and Sakon Nakhon province of Thailand, respectively. The pure cultures of *Aspergillus sojae* (TISTR3037) and *Lactobacillus delbrueckii* (*L. delbrueckii*) subsp. *lactis* were purchased from Thailand Institute of Scientific and Technological Research (TISTR). The extracts of rice bran were separately prepared. Firstly, rice bran sample was pasteurized in sterile distilled water (DW) at 63 ± 1 °C for 30 min. Then, rice bran was fermented by *Aspergillus sojae* (TISTR3037), which was used as starter culture at 30 °C for 2–5 days and then fermented by *L. delbrueckii* subsp. *lactis* for another 2–5 days. Afterward, the fermented sap was collected by centrifugation and vacuum filtration and dried using lyophilization (Martin Christ, Osterode am Harz, Germany) under the following conditions: freezing at −80 °C, followed by a primary freeze-drying at 0.4 mbar to eliminate free water (ending at 3 °C) and by two subsequent cycles of secondary freeze-drying with a pressure variation of 1.6 mbar at 0.001 mbar to remove the bound water (ending at 10 °C). Finally, the dried powder of nonfermented rice bran (H7NF and G13NF) and fermented rice bran (H7F and G13F) were kept at −20 °C until used in experiments. For the preparation of extracts, the powder of each rice bran (nonfermentation and fermentation) was dissolved in DW for the stock of the extracts and kept in −20 °C prior to use in further experiments. To investigate probiotic growth, antibacterial activity and cytotoxicity, the extracts were prepared in the diluents, including de Man–Rogaosa–Sharpe (MRS) broth, DW, and RPMI 1640 medium, respectively.

### 4.2. Chemical Composition Profile by Attenuated Total Reflection–Fourier Transform Infrared Spectroscopy (ATR-FTIR) 

The chemical compositions of both nonfermented and fermented rice bran extracts of H7 and G13 were investigated using ATR-FTIR (4500 Series, Agilent Technologies, Santa Clara, CA, USA). The spectra were recorded in the wave number range of 500–4000 cm^−1^ to manipulate the operating software of the ATR-FTIR instrument to transform the data into derivative spectra. 

### 4.3. Prebiotic Property on L. delbrueckii Growth Using Broth Dilution Technique and the Potential of Hydrogen Ion (pH) Measurement 

Various concentrations (3.125, 6.25, 12.5, 25 and 50 mg/mL) of fermented rice bran H7 (H7F) and G13 (G13F) extracts were cocultured with *L. delbrueckii* (2 × 10^4^ cfu/mL) in 27.6 g/L de Man–Rogaosa–Sharpe (MRS) broth under anaerobic conditions at 37 °C for 24 h. Bacterial numbers were analyzed by flow cytometric analysis (BD FACSCantoTM II flow cytometer, BD Biosciences, San Jose, CA, USA) employing counting beads calculation (BD Biosciences, CA, USA) followed with pH value measurement, compared to nonfermented rice bran (H7NF and G13NF) extracts, by using pH meter (Starter 3100, Ohaus, Parsippany, NJ, USA). 

### 4.4. Antimicrobial Activity Assays 

#### 4.4.1. Agar Well Diffusion and Broth Microdilution Technique 

Agar well diffusion test was employed to test the inhibitory effect of fermented and nonfermented rice bran extracts against pathogenic bacteria. Mueller–Hinton (MH) agars were drilled with 6 mm diameter cork-borer and then streaked with *Escherichia coli* (*E. coli*) ATCC25922 or *Staphylococcus aureus* (*S. aureus*) ATCC29213. A total of 100 μL of 400 mg/mL (excessive concentration dose) fermented (H7F and G13F) and nonfermented rice bran (H7NF and G13NF) extracts dissolved in DW were added in holes and then incubated at 37 °C for 24 h before determining the diameter of inhibition zone. Distilled water (DW) was used for noninhibition control, whereas bacteria cocultured with 5 μg/mL of Ampicillin (Amp) were used for inhibition control.

Moreover, the solution of fermented rice bran extracts was diluted into various concentrations, ranging between 200, 100, 50, 25, 12.5, 6.25, 3.12, 1.56, and 0.8 mg/mL, and used in the experiment to define a minimum inhibition concentration (MIC) as well as a minimum bactericidal concentration (MBC) by broth microdilution technique. The fermented rice bran extract solutions were cocultured with pathogenic bacteria in microtiter plate at 37 °C for 24 h, and subsequently, 10 μL of each was dropped onto MH agar and followed with a 24 h incubation. Finally, the colonies in each unit were observed. The colony numbers of the treated group were compared to that of noninhibition control (untreated group; distilled water (DW) + microbial + medium). The MIC was indicated at the lowest concentration, resulting in colony density and size reduction as evidenced by the bacterial growth inhibitory effect. In contrast, the MBC was accounted for at the lowest concentration, resulting in no bacterial colony formation [62].

#### 4.4.2. Biofilm Formation by Scanning Electron Microscopy (SEM)

*E. coli* and *S. aureus* were cultured in trypticase soy broth (TSB) in 6-well plates. The concentrations of fermented rice bran extract solution dissolved in TBS were adjusted according to MIC. Bacteria were incubated at 37 °C for 18 h before washing and fixation with 4% formaldehyde. Finally, samples were dehydrated with absolute ethanol and observed under a scanning electron microscope (SEM) (S-3000N Hitachi, Tokyo, Japan).

### 4.5. Safety and Toxicity Assays 

#### 4.5.1. Toxicity Test of Human Peripheral Blood Mononuclear Cells (PBMCs) in In Vitro Model

Human peripheral blood mononuclear cells (PBMCs) from the leftover buffy coat permitted by the Khon Kaen University Ethical Committee Review Board (HE651334) were cultured in RPMI 1640 media supplemented with 10% fetal bovine serum at 37 °C for 24 h with presenting of fermented rice bran extracts at various concentrations of 5, 10, 20, 25, and 50 mg/mL r DW for the control. Compared to the control, %viability of cell was analyzed by MTT assay.

#### 4.5.2. Study Population in In Vivo Model

A total of 160 Wistar rats, including 80 males and 80 females, aged between 6–8 weeks, were employed in this experiment. The animals were provided by the National Laboratory Animal Center, Mahidol University, and they were later treated at the Northeast Laboratory Animal Center, Khon Kaen University, Thailand. This study was approved by the Ethical Committee of Khon Kaen University (AEKKU-NELAC 7/2557), whose acute and chronic toxicity study was performed in respect to OECD guideline 420 [63] and OECD guideline 452 [64], respectively. 

#### 4.5.3. Acute Toxicity Test 

A total of 80 rats were used and divided into 8 groups with 10 rats, including 5 females and 5 males in each. Those in groups I, II, and III received an orally fed single dose of G13F for 14 days at 1000, 2500, and 5000 mg/kg/day; those in group IV, V, and VI received oral feeding of H7F in the same dose; while those in group VII and VIII were vehicle (DW) and untreated group, respectively. Each rat’s body weight was recorded daily, while the weight of organs, blood chemical values, hematological values, and histopathology of organs were examined after completion of a 14-day term of study.

#### 4.5.4. Chronic Toxicity Test 

Similar to the acute toxicity test, 80 rats were used and divided into 8 groups with 10 rats, including 5 females and 5 males, in each. For 6 months, the assigned groups I, II, and III daily received an orally fed dose of G13F at 500, 1000, and 2000 mg/kg/day; group IV, V, and VI received H7F; and VII and VIII groups were vehicle and untreated group, respectively. Each rat’s body weight was recorded weekly, while the weight of organs, blood chemical values, hematological values, and histopathology of organs were examined after 6-month term of treatment. 

### 4.6. Statistical Analysis 

The data are presented in mean ± standard deviation (SD) from at least triplicates of independent studied unit. The flow cytometry data were analyzed by the BD FACSDivas^TM^ software (BD Biosciences, Franklin, CA, USA). The statistical analysis was performed by SPSS (version 17, SPSS Inc., San Diego, IL, USA) and GraphPad Prism software (GraphPad Software Inc., Chicago, CA, USA). The nonparametric data were analyzed by Mann–Whitney U test. Statistical significance was considered at *p*-value less than 0.05.

## 5. Conclusions

The health benefits of fermented rice bran extracts from black rice cultivar (H7F) and germinated brown rice cultivar (G13F) have been evidenced. They exhibited capacity in controlling bacterial growth with different potencies: growth-promoting for beneficial bacteria via the prebiotic property, while inhibiting pathogenic bacterial growth by diminishing biofilm formation and promoting cell membrane breakage. In addition, their high degree of safety was demonstrated. Coincidently, the reduced cholesterol levels and modulating immune cell response were manifested. Conclusively, H7F and G13F are suggested as promising functional ingredients for health-promoting products. 

## Figures and Tables

**Table 1:** Effects on hematological values in male (**A**) and female Wistar rats (**B**) of fermented rice bran H7 (H7F) and G13 extracts (G13F) in chronic toxicity study.

**(A)** **Parameters**	**Male (*n* = 5 in each group)**							
	**Vehicle**	**Untreated**	**Doses of H7F (mg/kg)**			**Doses of G13F (mg/kg)**		
			**500**	**1000**	**2000**	**500**	**1000**	**2000**
RBC (10^6^/µL)	9.04 ± 0.49	8.75 ± 0.84	8.05 ± 2.04	8.80 ± 0.06	8.16 ± 1.42	9.16 ± 1.29	9.07 ± 0.61	9.04 ± 0.60
HGB (g/dL)	15.90 ± 0.32	14.96 ± 1.22	14.16 ± 3.57	15.55 ± 0.66	14.73 ± 2.55	15.88 ± 1.71	15.60 ± 0.58	15.60 ± 0.42
HCT (%)	48.26 ± 1.62	44.76 ± 3.78	41.04 ± 10.63	45.10 ± 2.16	42.18 ± 7.61	44.48 ± 4.38	43.96 ± 1.59	44.38 ± 1.62
WBC (10^3^/µL)	3.16 ± 1.63	2.43 ± 1.80	3.71 ± 1.78	4.63 ± 1.59	4.15 ± 1.39	5.03 ± 1.69	**6.08 ± 1.08 ^a,b^**	**6.13 ± 1.01 ^a,b^**
PLT (10^3^/µL)	502.8 ± 327.2	482.0 ± 156.1	587.6 ± 193.2	565.0 ± 235.4	558.8 ± 378.4	**838.6 ± 120.2 ^b^**	**719.0 ± 99.4 ^b^**	**826.8 ± 124.2 ^b^**
MPV (fL)	8.0 ± 0.30	7.6 ± 0.10	7.7 ± 0.36	7.7 ± 0.14	7.6 ± 0.31	7.6 ± 0.20	7.4 ± 0.13	7.5 ± 0.11
NE (%)	15.62 ± 3.64	22.82 ± 18.06	17.70 ± 8.87	15.48 ± 4.67	12.23 ± 3.35	15.42 ± 4.41	12.14 ± 3.08	12.18 ± 2.25
LY (%)	76.42 ± 5.46	70.80 ± 15.27	77.52 ± 7.35	77.48 ± 3.49	83.50 ± 2.65	77.72 ± 4.07	83.50 ± 3.36	82.80 ± 4.03
MO (%)	4.88 ± 3.66	4.16 ± 2.42	2.84 ± 1.80	3.63 ± 1.56	3.28 ± 1.67	5.14 ± 0.42	2.90 ± 0.88	3.68 ± 1.99
EO (%)	3.04 ± 3.33	2.12 ± 1.90	1.86 ± 1.23	3.23 ± 1.9	0.93 ± 0.25	1.66 ± 0.50	1.44 ± 0.30	1.28 ± 0.33
BA (%)	0.00 ± 0.00	0.10 ± 0.14	0.08 ± 0.11	0.00 ± 0.00	0.00 ± 0.00	0.06 ± 0.13	0.02 ± 0.05	0.06 ± 0.09
MCV (fL)	53.44 ± 1.17	51.16 ± 0.65	50.92 ± 0.87	51.50 ± 2.23	51.73 ± 2.73	48.76 ± 1.94	48.54 ± 1.12	49.22 ± 2.40
MCH (pg)	17.62 ± 0.63	17.10 ± 0.28	17.58 ± 0.28	17.75 ± 0.65	18.05 ± 0.31	17.40 ± 0.55	17.22 ± 0.55	17.30 ± 0.81
MCHC (g/dL)	32.98 ± 0.46	33.42 ± 0.24	34.54 ± 0.58	34.48 ± 0.49	34.95 ± 1.49	35.68 ± 0.40	35.50 ± 0.37	35.18 ± 0.56
RDW (%)	21.88 ± 0.84	21.76 ± 1.22	20.20 ± 3.43	20.88 ± 0.41	20.70 ± 2.98	22.42 ± 2.24	21.92 ± 1.20	22.32 ± 1.18
**(B)** **Parameters**	**Female (*n* = 5 in each group)**							
	**Vehicle**	**Untreated**	**Doses of H7F (mg/kg)**			**Doses of G13F (mg/kg)**		
			**500**	**1000**	**2000**	**500**	**1000**	**2000**
RBC (10^6^/µL)	8.06 ± 1.78	7.57 ± 1.10	8.37 ± 0.34	8.19 ± 0.30	7.94 ± 1.06	8.04 ± 0.33	7.72 ± 0.76	8.33 ± 0.35
HGB (g/dL)	15.30 ± 2.69	13.92 ± 1.96	15.44 ± 0.71	15.28 ± 0.50	14.62 ± 2.02	15.00 ± 0.86	14.10 ± 1.57	15.56 ± 0.72
HCT (%)	43.62 ± 8.36	41.14 ± 5.50	44.06 ± 2.35	43.86 ± 1.59	42.56 ± 5.50	43.26 ± 2.32	41.00 ± 40.00	44.66 ± 1.85
WBC (10^3^/µL)	1.85 ± 0.67	1.97 ± 1.77	2.83 ± 1.71	2.90 ± 0.89	**3.27 ± 0.98 ^a^**	**3.46 ± 1.02 ^a^**	2.26 ± 0.68	2.96 ± 1.17
PLT (10^3^/µL)	514.6 ± 283.2	484.4 ± 402.4	560.0 ± 49.7	575.4 ± 235.4	568.8 ± 238.2	682.2 ± 133.4	722.0 ± 91.5	703.8 ± 110.8
MPV (fL)	7.8 ± 0.54	7.4 ± 0.28	7.3 ± 0.3	7.4 ± 0.23	7.4 ± 0.48	7.4 ± 0.15	7.5 ± 0.23	7.4 ± 0.13
NE (%)	14.87 ± 7.74	12.62 ± 7.00	8.52 ± 2.25	7.45 ± 2.95	12.42 ± 2.79	14.80 ± 9.93	20.23 ± 5.24	11.78 ± 3.41
LY (%)	76.53 ± 6.74	80.52 ± 11.81	85.20 ± 7.12	82.12 ± 3.74	76.90 ± 8.88	81.48 ± 9.62	75.50 ± 3.47	82.86 ± 5.38
MO (%)	3.73 ± 0.84	2.00 ± 1.59	2.90 ± 1.72	4.68 ± 1.53	5.26 ± 3.79	2.52 ± 1.56	2.23 ± 1.72	4.12 ± 2.20
EO (%)	4.86 ± 4.80	4.76 ± 4.52	3.34 ± 4.02	4.40 ± 6.53	5.30 ± 3.34	1.16 ± 0.49	2.03 ± 0.32	1.30 ± 0.60
BA (%)	0.82 ± 1.65	0.10 ± 0.14	0.04 ± 0.09	0.54 ± 1.00	0.12 ± 0.16	0.04 ± 0.09	0.00 ± 0.00	0.12 ± 0.16
MCV (fL)	54.38 ± 1.61	54.50 ± 1.90	52.64 ± 1.64	53.58 ± 1.60	53.64 ± 0.17	53.82 ± 0.99	53.17 ± 0.96	53.68 ± 2.16
MCH (pg)	19.36 ± 4.04	18.40 ± 0.38	18.46 ± 0.43	18.66 ± 0.35	18.42 ± 0.71	18.64 ± 0.45	18.23 ± 0.35	18.70 ± 0.59
MCHC (g/dL)	35.52 ± 6.82	33.82 ± 0.96	35.08 ± 0.40	34.86 ± 0.54	34.34 ± 0.49	34.70 ± 0.25	34.37 ± 0.55	34.82 ± 0.47
RDW (%)	18.48 ± 2.75	17.38 ± 2.12	18.52 ± 1.10	18.78 ± 0.55	18.28 ± 1.57	18.04 ± 1.27	18.37 ± 0.84	19.00 ± 0.55

Data are represented as mean ± standard deviation (SD). N = number of samples; RBC = red blood cell; HGB = hemoglobin; HCT = hematocrit; WBC = white blood cell; PLT = platelet; MPV = mean platelet volume; NE = neutrophils; LY = lymphocyte; MO = monocyte; EO = eosinophils; BA = basophile; MCV = mean cell volume; MCH = mean corpuscular hemoglobin; MCHC = mean corpuscular hemoglobin concentration; RDW = red blood cell distribution width; statistical significance at *p*-value < 0.05 when compared to vehicle group (A) and untreated group (B).

**Table 2:** Effects on biochemical parameters in male (**A**) and female Wistar rats (**B**) of fermented rice bran H7 (H7F) and G13 extracts (G13F) in chronic toxicity study.

**(A)** **Parameters**	**Male** **(*n* = 5 in each group)**							
	**Vehicle**	**Untreated**	**Doses of H7F (mg/kg)**			**Doses of G13F (mg/kg)**		
			**500**	**1000**	**2000**	**500**	**1000**	**2000**
BUN (mg/dL)	20.34 ± 0.78	26.32 ± 2.84	23.36 ± 1.62	17.50 ± 1.10	20.23 ± 2.63	26.3 ± 0.55	24.58 ± 0.40	25.48 ± 0.95
Cr (mg/dL)	0.32 ± 0.03	0.31 ± 0.06	0.30 ± 0.02	0.29 ± 0.05	0.27 ± 0.04	0.28 ± 0.29	0.31 ± 0.02	0.27 ± 0.03
Na (mmol/L)	145.6 ± 2.97	145.0 ± 1.00	142.0 ± 2.12	144.8 ± 1.71	144.5 ± 1.92	141.2 ± 1.92	142.2 ± 0.84	141.8 ± 2.28
K (mmol/L)	5.89 ± 0.26	5.60 ± 0.12	5.72 ± 0.58	5.69 ± 0.27	5.52 ± 0.70	6.06 ± 0.85	5.58 ± 0.33	6.35 ± 1.03
Cl (mmol/L)	97.3 ± 3.30	97.3 ± 2.32	96.0 ± 1.66	98.8 ± 1.50	99.5 ± 0.93	97.8 ± 1.35	97.7 ± 1.04	97.2 ± 1.88
HCO_3_- (mmol/L)	24.8 ± 3.35	27.8 ± 1.30	26.4 ± 2.19	27.5 ± 0.58	26.0 ± 0.82	27.8 ± 1.64	28.8 ± 1.30	26.4 ± 0.55
Cholesterol (mg/dL)	113.8 ± 25.28	90.8 ± 7.79	**74.8 ± 8.87 ^a,b^**	**81.5 ± 11.27**	**76.2 ± 6.34 ^a,b^**	90.8 ± 9.01	**78.6 ± 5.32 ^a,b^**	81.6 ± 14.77
Triglyceride (mg/dL)	235.0 ± 82.85	181.8 ± 31.15	150.2 ± 34.27	175.7 ± 40.96	185.8 ± 55.87	182.4 ± 32.55	174.2 ± 42.10	167.2 ± 38.47
Total protein (g/dL)	6.66 ± 0.43	6.42 ± 0.18	6.32 ± 0.30	6.25 ± 0.17	6.38 ± 0.34	6.22 ± 0.34	6.16 ± 0.17	6.38 ± 0.32
Albumin (g/dL)	4.16 ± 0.40	4.32 ± 0.13	4.04 ± 0.25	4.23 ± 0.22	4.23 ± 0.29	4.02 ± 0.26	4.10 ± 0.12	4.22 ± 0.23
Total bilirubin (mg/dL)	0.08 ± 0.02	0.07 ± 0.01	0.08 ± 0.02	0.10 ± 0.03	0.10 ± 0.02	0.09 ± 0.29	0.08 ± 0.02	0.07 ± 0.02
ALT (U/L)	76.6 ± 55.32	99.6 ± 21.78	66.4 ± 25.23	44.2 ± 16.64	47.2 ± 5.74	73.8 ± 19.31	86.4 ± 48.52	55.2 ± 12.09
AST (U/L)	121.6 ± 39.87	162.0 ± 31.68	109.6 ± 15.63	147.2 ± 30.87	143.0 ± 23.02	136.2 ± 39.15	135.6 ± 20.31	87.4 ± 26.54
ALP (U/L)	97.8 ± 16.68	79.0 ± 19.33	79.2 ± 16.45	62.0 ± 8.76	94.0 ± 28.04	98.2 ± 26.78	74.4 ± 5.77	69.8 ± 6.50
**(B)** **Parameters**	**Female (*n* = 5 in each group)**							
	**Vehicle**	**Untreated**	**Doses of H7F (mg/kg)**			**Doses of G13F (mg/kg)**		
			**500**	**1000**	**2000**	**500**	**1000**	**2000**
BUN (mg/dL)	20.48 ± 1.86	19.26 ± 1.86	12.38 ± 1.73	21.12 ± 3.78	15.04 ± 3.25	26.94 ± 1.66	26.77 ± 5.33	24.64 ± 2.29
Cr (mg/dL)	0.32 ± 0.01	0.33 ± 0.07	0.33 ± 0.05	0.35 ± 0.05	0.31 ± 0.05	0.33 ± 0.03	0.25 ± 0.02	0.34 ± 0.05
Na (mmol/L)	141.4 ± 1.14	145.4 ± 1.34	143.2 ± 1.30	141.6 ± 1.50	141.8 ± 2.59	141.2 ± 1.30	141.0 ± 0.70	144.0 ± 1.00
K (mmol/L)	4.93 ± 0.36	5.07 ± 0.33	4.84 ± 0.23	5.22 ± 0.16	5.79 ± 0.94	5.87 ± 0.22	5.66 ± 0.59	5.46 ± 0.17
Cl (mmol/L)	98.3 ± 0.66	100.9 ± 1.22	102.8 ± 0.89	98.9 ± 1.64	101.7 ± 1.90	98.7 ± 1.14	100.0 ± 1.83	100.8 ± 1.51
HCO_3_- (mmol/L)	28.2 ± 0.84	26.2 ± 1.30	26.2 ± 1.30	26.6 ± 0.89	24.8 ± 3.42	27.4 ± 1.82	27.0 ± 1.40	27.0 ± 1.23
Cholesterol (mg/dL)	80.6 ± 13.85	79.8 ± 18.42	80.2 ± 18.31	84.4 ± 15.24	89.2 ± 19.72	82.6 ± 8.79	99.3 ± 33.56	89.4 ± 21.13
Triglyceride (mg/dL)	187.2 ± 49.78	166.4 ± 26.12	125.8 ± 40.68	152.4 ± 45.10	128.4 ± 49.92	139.2 ± 35.27	124.3 ± 57.29	153.8 ± 50.49
Total protein (g/dL)	6.40 ± 0.17	6.62 ± 0.61	6.08 ± 0.15	6.40 ± 0.43	6.24 ± 0.40	6.28 ± 0.32	6.57 ± 0.35	6.36 ± 0.21
Albumin (g/dL)	4.48 ± 0.11	4.58 ± 0.35	4.38 ± 0.13	4.44 ± 0.33	4.34 ± 0.23	4.58 ± 0.26	4.83 ± 0.15	4.60 ± 0.14
Total bilirubin (mg/dL)	0.07 ± 0.02	0.07 ± 0.02	0.09 ± 0.02	0.08 ± 0.03	0.09 ± 0.02	0.06 ± 0.01	0.07 ± 0.04	0.04 ± 0.01
ALT (U/L)	41.0 ± 7.45	42.2 ± 21.98	34.6 ± 18.19	40.4 ± 7.37	30.6 ± 6.19	43.2 ± 0.09	45.0 ± 2.20	44.6 ± 3.58
AST (U/L)	113.0 ± 19.53	126.0 ± 47.21	57.0 ± 21.24	86.2 ± 13.95	71.8 ± 21.23	114.2 ± 28.72	98.0 ± 29.10	115.0 ± 42.86
ALP (U/L)	34.6 ± 7.16	33.4 ± 8.96	22.0 ± 1.52	32.2 ± 4.49	30.0 ± 13.29	58.4 ± 17.14	47.0 ± 10.58	59.8 ± 29.94

Data are represented as mean ± standard deviation (SD). N = number of samples; BUN = blood urea nitrogen; Cr = creatinine; Na = sodium; K = potassium; Cl = chloride; HCO_3_^−^ = bicarbonate; ALT = alanine aminotransferase; AST = aspartate aminotransferase; ALP = alkaline phosphatase; statistical significance at *p*-value < 0.05 when compared to vehicle group (A) and untreated group (B).

## Data Availability

Data is contained within the article.
